# The effects of acupuncture on patients with premature ovarian insufficiency and polycystic ovary syndrome: an umbrella review of systematic reviews and meta-analyses

**DOI:** 10.3389/fmed.2024.1471243

**Published:** 2024-11-25

**Authors:** Tianyu Bai, Xinyun Deng, Jieyu Bi, Linlin Ni, Zhaohui Li, Xiumei Zhuo

**Affiliations:** ^1^Department of Acupuncture-Moxibustion and Tuina, Shandong Provincial Third Hospital, Shandong University, Jinan, Shandong, China; ^2^Center for Reproductive Medicine, Zibo Maternal and Child Health Hospital, Zibo, Shandong, China; ^3^Center for Reproductive Medicine, The Second Affiliated Hospital of Shandong University of Traditional Chinese Medicine, Jinan, Shandong, China; ^4^Integrated Chinese and Western Medicine Assisted Pregnancy Clinic, Hohhot First Hospital, Hohhot, Inner Mongolia, China; ^5^Department of Acupuncture-Moxibustion and Tuina, Gaomi Maternity and Child Health Hospital, Gaomi, Shandong, China

**Keywords:** acupuncture, polycystic ovary syndrome, premature ovarian insufficiency, premature ovarian failure, primary ovarian insufficiency, pregnancy rate, umbrella meta-analysis

## Abstract

**Background:**

Previous studies have suggested that acupuncture could improve the clinical outcomes of women with premature ovarian insufficiency (POI) and polycystic ovary syndrome (PCOS). However recent meta-analyses have provided inconclusive findings. This umbrella meta-analysis aimed to explore the effect of acupuncture therapies on PCOS and POI outcomes.

**Methods:**

A systematic literature search was carried out in in PubMed, Scopus, Web of Science, and Chinese databases, including Wan Fang Data Knowledge Service Platform, CBM, CNKI, and VIP up until April 2024 to gather relevant studies. Inclusion criteria were meta-analyses on the effect of acupuncture or combined therapies with standard medications or traditional Chinese medicine (TCM) on PCOS and POI. The outcomes were pregnancy rates, ovulation rates, hormone levels, glycemic indices, resumption of menstruation, endometrial thickness, live birth rates, abortion rates, and body mass index (BMI). Studies with irrelevant interventions, animal studies, reviews without quantitative analysis, and studies with insufficient data were excluded. Standardized mean difference (SMD) with a 95% confidence interval (CI) and relative risk (RR) with a 95% CI were used as effect sizes to pool the data using a random effects model.

**Results:**

A total of 38 meta-analyses, 20 studies (sample size: 27,106 patients) for PCOS and 18 studies (sample size: 19,098 patients) for POI, were included. Overall, in women with PCOS, acupuncture therapies were significantly associated with a higher pregnancy rate, ovulation rate, and reduced serum levels of luteinizing hormone (LH), testosterone, LH/follicle-stimulating hormone (FSH), insulin resistance, and BMI. Moreover, FSH, fasting glucose, and fasting insulin levels were improved in subgroup analyses. For POI, acupuncture significantly improved serum levels of LH, FSH, LH/FSH ratio, and estradiol.

**Conclusion:**

Acupuncture-related therapies improve pregnancy rate, and metabolic and hormonal imbalances in patients with POI and PCOS.

**Systematic review registration:**

The protocol of the study was registered in PROSPERO (CRD42024572893). Available from: https://www.crd.york.ac.uk/prospero/display_record.php?ID=CRD42024572893.

## Introduction

Polycystic ovary syndrome (PCOS) and premature ovarian insufficiency (POI) are two distinct complex endocrine disorders that potentially interfere with women’s reproductive health and fertility (1). PCOS and POI impact 10 and 1% of women in their reproductive years, respectively, and their prevalence is increasing globally (2). PCOS is the leading cause of hyperandrogenism, oligoanovulation, and infertility in women, featured by hormonal imbalances, irregular menstrual cycles, and the presence of polycystic ovaries (3). Women with PCOS may encounter insulin resistance, as well as issues like obesity, inflammation, acne, hair thinning, mood fluctuations, and excessive hair growth (4). The pathophysiology of PCOS involves elevated levels of gonadotropin-releasing hormone (GnRH), which disrupt the normal LH/FSH ratio, potentially causing early luteinization of granulosa cells and impairing follicular development (5). POI, on the other side, is featured by the cessation of ovarian function before the age of 40, leading to hypergonadotropic amenorrhea, elevated gonadotrophins, and low estradiol (E2) levels (6). Although PCOS and POI have different pathophysiology, autoimmune disorders, genetic factors, and environmental factors have all been implicated in their development (7, 8). The complications of both conditions extend beyond fertility challenges; they pose long-term health risks such as type 2 diabetes, cardiovascular diseases, and osteoporosis (9, 10).

Polycystic ovary syndrome can be managed by herbal formulations, following a healthy lifestyle, drugs, acupuncture, and bariatric surgery (4). To restore ovulation and improve fertility, current pharmacological treatments for PCOS include letrozole, an aromatase inhibitor, as a first-line therapy, with clomiphene citrate serving as an alternative when initial treatments fail (11). The combination of clomiphene citrate with metformin has shown promise for women who do not respond to clomiphene alone. This approach addresses both ovulation induction and insulin resistance, a common issue in PCOS (12). Newer medications targeting insulin sensitivity, such as incretin mimetics and Sodium/glucose cotransporter 2 (SGLT2) inhibitors, have emerged as effective options for managing metabolic dysfunction in PCOS, potentially reducing cardiovascular risks (13). Emerging evidence suggests that inositol and vitamin D supplementation may improve metabolic parameters in women with PCOS, although further research is needed to establish clear guidelines (14). Moreover, emphasis on lifestyle modifications, such as weight loss and increased physical activity, has been recommended as a critical component of managing PCOS. These changes can enhance metabolic health and improve reproductive outcomes (15). While these treatments are generally effective, concerns arise regarding adverse effects, including elevated risk of multifetal pregnancies, as well as patient adherence due to the extended duration of treatment necessary for individuals with PCOS (16).

For POI, HRT remains the cornerstone of POI management, focusing on estrogen repletion to alleviate symptoms and protect long-term health. Transdermal patches or transvaginal applications are preferred due to lower risks of venous thromboembolism compared to oral estrogen (17). However, HRT effectively addresses symptoms related to hormonal imbalances, it does not restore ovarian function or improve fertility outcomes. Additionally, a remarkable relapse rate of low estrogen symptoms is commonly observed following the cessation of the medication (18). Moreover, HRT has been linked to several serious adverse effects, such as an elevated risk of heart attack, breast cancer, and stroke (19). Oocyte donation continues to be the most effective assisted reproductive technology for women with POI, achieving pregnancy rates of 70–80%. However, ongoing research is exploring additional methods to enhance fertility in this population (20). Also, *in vitro* activation is an innovative technique that disrupts signaling pathways to activate dormant follicles in women with POI, showing potential for restoring ovarian function and improving fertility outcomes (21). Moreover, emerging biological therapies such as platelet-rich plasma (PRP) therapy, stem cell therapy, exosome therapy, and mitochondrial targeting therapies have offered new avenues for treating POI to activate oogenesis and improve the ovarian microenvironment (22).

Given these limitations in conventional treatments for PCOS and POI, alternative therapeutic approaches are being explored. Acupuncture a key component of traditional Chinese medicine, has gained attention as a potential alternative treatment for managing the symptoms of PCOS and POI because of its convenience and low side effects. Its use in treating reproductive disorders has shown promising results in improving menstrual regularity, hormone levels, metabolic factors, and overall fertility outcomes. Nevertheless, recent meta-analyses have provided contradictory results with positive (23, 24) or null (25) effects and the available evidence is insufficient to conclusively determine the effectiveness of acupuncture on critical reproductive outcomes in women with PCOS and POI. Differences in acupuncture methods, sample sizes, and treatment protocols may account for the heterogeneity reported in the findings of previous studies. This umbrella meta-analysis aimed to examine the effectiveness of acupuncture in addressing the symptoms of PCOS and POI by synthesizing data from multiple meta-analyses.

The term “premature ovarian insufficiency” (POI) has evolved significantly over time. In the 1940s, endocrinologist Fuller Albright first introduced the concept of “primary ovarian insufficiency (26).” Subsequently, the term “premature ovarian failure” (POF) became widely adopted. However, clinical practice has revealed that POF is a terminal stage of ovarian dysfunction, making its use as a universal diagnostic label inappropriate. In 2016, the European Society of Human Reproduction and Embryology (ESHRE) updated the terminology to “premature ovarian insufficiency” (POI) (27). This change underscored the early onset of ovarian function decline. In 2020, the International Menopause Society (IMS) released a white paper on POI, which continues to be referenced today (28). Therefore, this article will encompass all these terms as relevant to the discussion.

## Methods

This umbrella meta-analysis adhered to the guidelines outlined in the Preferred Reporting Items for Systematic Reviews and Meta-analyses (PRISMA) (26). The study was registered in PROSPERO [CRD42024572893], available from https://www.crd.york.ac.uk/prospero/display_record.php?ID=CRD42024572893.

### Search strategy

A systematic literature search was conducted in PubMed, Scopus, Web of Science, Wan Fang Data Knowledge Service Platform, Chinese Biomedical Database (CBM), Chinese National Knowledge Infrastructure (CNKI), and Journal Integration Platform (VIP) databases up to April 2024 to obtain pertinent studies. The search strategy encompassed the following terms: (“Acupuncture”[Mesh] OR “Acupuncture Therapy”[Mesh] OR “Electric Stimulation”[Mesh] OR “Electric Stimulation Therapy”[Mesh] OR acupuncture OR electrical stimulation* OR electro stimulation* OR electric stimulation* OR electrostimulus OR needl* OR Electro acupuncture OR Electroacupuncture) AND (“Polycystic Ovary Syndrome”[Majr] OR “Primary Ovarian Insufficiency”[Majr] OR “polycystic ovary syndrome” OR “polycystic ovar* syndrom*” OR “polycystic ovar* disease” OR PCOS OR “primary ovarian insufficiency” OR early menopause OR “premature menopause” OR “premature ovarian insufficiency” OR “premature ovarian failure” OR amenorrhea OR POI OR POF) AND (meta-analysis OR meta analysis). No language restrictions were considered during the search process. In addition, the list of the references of the obtained reviews was screened manually to prevent overlooking any relevant studies. We did not include registered websites or gray literature in the meta-analysis.

### Eligibility criteria

Two researchers individually assessed the identified publications, reviewed the titles/abstracts, and subsequently removed duplicate and irrelevant studies. Any disagreements were addressed through discussions and reaching a consensus. Eligible studies were then identified based on the predetermined inclusion and exclusion criteria. Meta-analyses of randomized clinical trials (RCT) meeting the following criteria were included: (1) participants: women diagnosed with PCOS or POI, (2) intervention: the intervention group used acupuncture or its combined therapy with standard medications or traditional Chinese medicine (TCM), (3) comparator: the control group received placebo/sham acupuncture or standard treatments, (4) outcomes: the primary outcome was pregnancy rate. Secondary outcomes included ovulation rates, hormone levels, such as luteinizing hormone (LH), estradiol (E2), follicle stimulating hormone (FSH), testosterone, LH/FSH ratio, glycemic indices, resumption of menstruation, endometrial thickness, live birth, abortion rate, and body mass index (BMI) after treatment for PCOS or POI. We included studies in which the mean and 95% confidence interval (CI) for continuous outcomes and relative risk (RR) with 95% CI for dichotomous outcomes were presented. Exclusion criteria were as follows: (1) subjects were treated with other interventions, (2) animal studies, (3) review studies with no quantitative analysis, and (4) studies that their data were not extractable.

### Data extraction

Data extraction was conducted independently by two researchers and subsequently reviewed by another researcher to gather relevant information potentially linked to the research outcomes, as outlined below: First author, country, publication year, outcome measures, sample size, number of the included studies, risk of bias (ROB) assessment, type of intervention, type of control, and effect sizes for the outcomes. In cases of missing data, we contacted to the corresponding authors of the relevant studies to request the necessary information. This approach allowed us to obtain the required data directly from the original sources.

### Quality evaluation

The quality of the included studies was evaluated using a critical appraisal tool for systematic reviews-2 (AMSTAR-2), which involves a structured approach to evaluate the reliability and rigor of systematic reviews, helping to determine the trustworthiness and validity of the study findings (27). The tool consists of 16 items divided into seven critical and nine non-critical domains that assesses various aspects of systematic reviews. The critical items evaluate aspects such as whether *a priori* protocols were established, the comprehensiveness of literature searches, justification for study exclusions, risk of bias assessments, appropriate meta-analysis methods, and consideration of publication bias. The non-critical items address additional factors like funding sources and potential conflicts of interest. The quality of studies were categorizes as very low, low, moderate, and high based on the AMSTAR-2 criteria. Two independent reviewers assessed each study using the AMSTAR-2 checklist. Any disagreements between reviewers were resolved through discussion to reach a consensus. We did not employ any automated tools in this process, as we aimed to ensure thorough and careful consideration of quality evaluation.

### Statistical analysis

Continuous data were analyzed using the standardized mean difference (SMD) and 95% CI, whereas binary data were assessed using the RR and 95% CI. The *I*^2^ test was employed to evaluate statistical heterogeneity. An *I*^2^ value exceeding 50% suggests the presence of significant heterogeneity. Due to expected heterogeneity, the data were combined using a random effects model. The origin of heterogeneity was investigated through subgroup analyses based on the quality of studies, type of intervention, duration of follow-up, and sample size. For outcomes with more than 10 effect sizes, meta-regression analysis was also conducted to assess the effect of the percentage of primary studies with low ROB on the pooled effect sizes. Sensitivity analyses were performed to assess the reliability of the analyses. If more than 10 effect sizes were available, publication bias was evaluated with the use of the funnel plot and Egger’s test (28). When there was a significant publication bias, the trim-and-fill method (29) was applied to adjust the results for the observed publication bias. The analysis was conducted using the Stata software version 13.

## Results

### Characteristics of the studies

**Figure 1:**
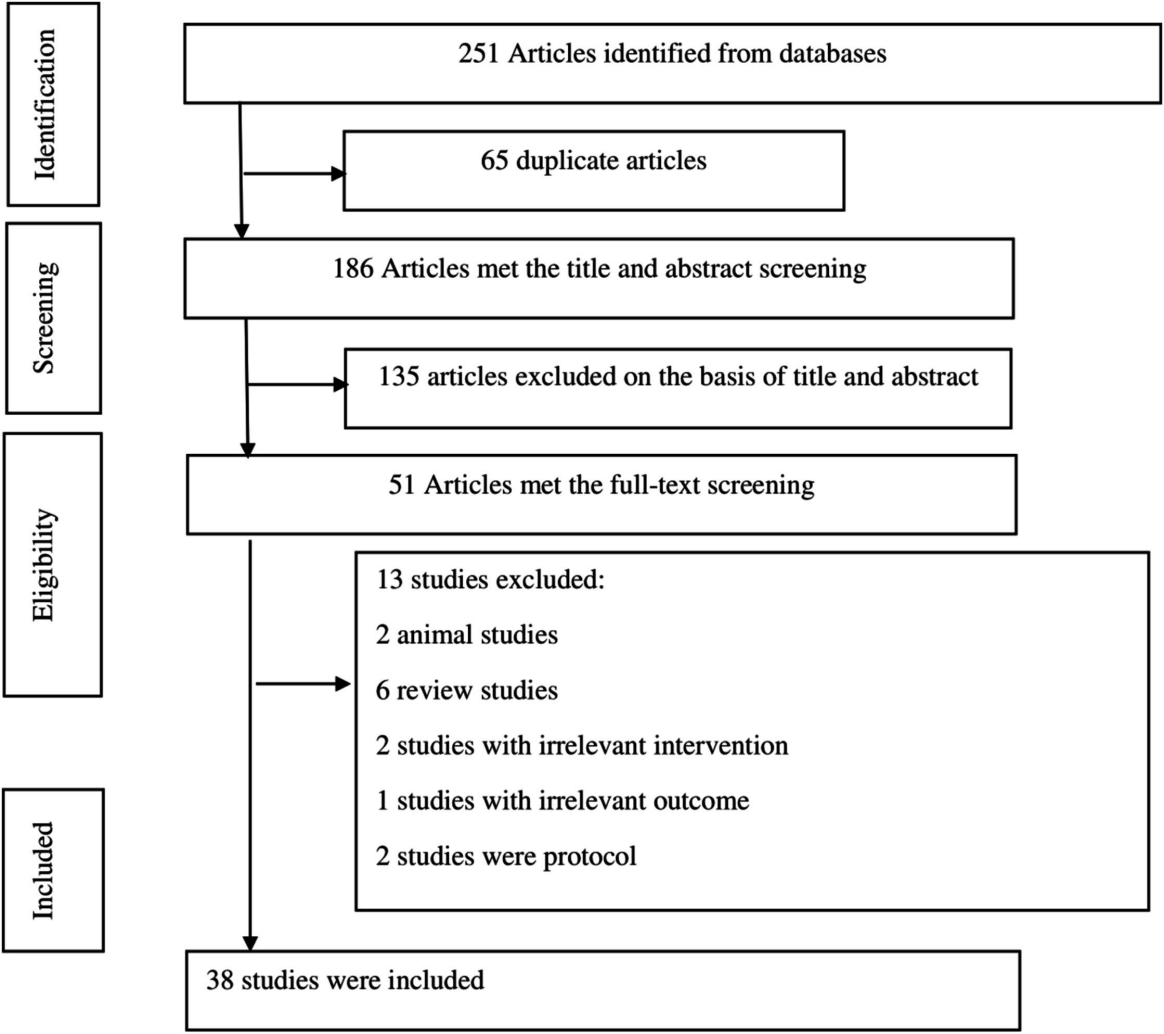
Flow diagram of the study.

Through a systematic search of databases, 251 articles were identified. Among these, 65 duplicates were removed, 135 publications were excluded following title and abstract screening, and 13 studies were excluded after a full-text assessment based on the inclusion/exclusion criteria. Excluded studies were review studies, protocols, animal studies, or studies with irrelevant exposure/outcome. The process of study selection is reported in Figure 1. Ultimately, 38 studies (23–25, 30–64), published between 2014 and 2023, were included in the umbrella meta-analysis. There were 20 studies (sample size: 27,106 patients) on PCOS (24, 25, 30–33, 37, 38, 40, 42, 44, 48, 50, 52, 55, 56, 60–62, 64) and 18 studies (sample size: 19,098 patients) on POI (23, 34–36, 39, 41, 43, 45–47, 49, 51, 53, 54, 57–59, 63). The sample sizes of the studies varied from 368 to 3,231 participants. The mean age of patients was between 23.98 to 35.96 years. The interventions in the treatment group included various types of acupuncture (needle acupuncture, electroacupuncture, auricular points, acupoint catgut embedding, and warm acupuncture), acupuncture with other therapies (TCM or standard medications), acupuncture with moxibustion, moxibustion, and moxibustion with TCM. The control groups received shame acupuncture or standard medications plus TCM. The Follow-up duration of studies ranged from 2.5 to 5.7 months. The ROB in the studies was evaluated utilizing either the Cochrane tool (65) or Jadad score (66). The included meta-analyses exhibited significant diversity in the proportion of RCTs with low ROB, ranging from 0 to 75% of the included RCTs demonstrating low ROB. For PCOS outcomes, pregnancy rate was reported in 12 studies, ovulation rate in 7 studies, resumption of menstruation in 2 studies, rate of live birth in 3 studies, abortion rate in 2 studies, serum LH in 9 studies, serum FSH in 4 studies, LH/FSH ratio in 6 studies, serum testosterone in 7 studies, endometrial thickness in 2 studies, fasting plasma glucose (FPG) in 3 studies, oral glucose tolerance test (OGTT) in 2 studies, fasting insulin in 4 studies, homeostatic model assessment for insulin resistance (HOMA-IR) in 3 studies, and BMI in 6 studies. For POI outcomes, serum LH was presented in 15 studies, serum FSH in 17 studies, and serum estradiol (E2) in 15 studies. The characteristics of the included studies are presented in Table 1.

**Table 1:** Characteristics of the included studies.

Study	Year	Country	Type of disease	Included studies	Mean age	Sample size	Intervention type	Control	Risk of bias assessment, high quality/total studies	High quality studies (%)	Follow-up duration (month)	Outcomes	Quality
Lim (62)	2016	Australia	PCOS	5	NR	413	Acupuncture	Shame acupuncture/Medications	Cochrane tool, 1/5	20	2.5	Pregnancy rate, resumption of menstruation	High
Lim (50)	2019	Australia	PCOS	8	NR	1,546	Acupuncture	Shame acupuncture/Medications	Cochrane tool, 1/8	12	NR	Pregnancy rate, ovulation rate, live birth, abortion	High
Chen (24)	2022	China	PCOS	9	27.44	1,159	Acupuncture plus medications	Medications	Cochrane tool, 1/9	11	3.8	Pregnancy rate, ovulation rate, HOMA-IR	Moderate
Ma (64)	2014	China	PCOS	6	NR	587	Acupuncture	Medications	Cochrane tool, 0/6	0	3	ovulation rate, testosterone	Low
Jo (55)	2017	Republic of Korea	PCOS	4	29.75	430	Acupuncture	Medications	Cochrane tool, 3/4	75	NR	Pregnancy rate, live birth	Moderate
Zheng (38)	2021	China	PCOS	10	24.71	737	Acupuncture	Medications	Cochrane tool, 5/10	50	4.62	HOMA-IR, BMI, FPG, OGTT	Moderate
Wu (44)	2020	China	PCOS	22	27.88	2,315	Acupuncture Acupuncture plus medications	Shame acupuncture/Medications	Cochrane tool, 14/22	63	3.9	Pregnancy rate, ovulation rate, LH, testosterone, live birth, LH/FSH ratio	Moderate
Jo (56)	2017	Korea	PCOS	28	23.98	2093	Acupuncture Acupuncture plus medications	Shame acupuncture/Medications	Cochrane tool, 6/28	21	3.2	Pregnancy rate, LH, testosterone, LH/FSH ratio, fasting insulin	High
Li (37)	2022	China	PCOS	25	35.38	1991	Acupuncture plus Moxibustion	Shame acupuncture/Medications	Cochrane tool, 0/25	0	4.28	Pregnancy rate, ovulation rate, LH, testosterone, LH/FSH ratio, FSH, abortion, fasting insulin, BMI	High
Liang (33)	2023	China	PCOS	47	NR	3,537	Acupuncture plus TCM	Shame acupuncture/Medications plus TCM	Cochrane tool, 19/47	40	NR	Pregnancy rate, ovulation rate, LH, testosterone, LH/FSH ratio, BMI	Moderate
Yun (48)	2019	China	PCOS	22	20–40 years	2,591	Acupuncture plus medications Acupuncture plus TCM and Medications	Medications	Cochrane tool, 0/22	0	NR	Pregnancy rate, ovulation rate, LH, LH/FSH ratio, endometrial thickness	Moderate
Qu (61)	2016	China	PCOS	9	NR	531	Acupuncture Acupuncture plus TCM	Shame acupuncture/Medications plus TCM	Cochrane tool, 2/9	22	NR	LH, LH/FSH ratio, FSH, fasting insulin, BMI, FPG	Moderate
Hu (40)	2021	China	PCOS	13	29.5	1,297	Acupuncture plus medications	Medications	Cochrane tool, 6/13	46	NR	LH	Moderate
Liu (32)	2022	China	PCOS	7	26.76	728	Electroacupuncture Acupuncture plus Medications Acupuncture	Shame acupuncture/Medications	Cochrane tool, 3/7	42	4.6	HOMA-IR, fasting insulin, BMI, FPG, OGTT	Moderate
Chao-chao	2017	China	PCOS	25	NR	1,636	Acupoint catgut embedding	Medications	Cochrane tool, 0/32pcos	0	NR	Testosterone, resumption of menstruation	Moderate
Meizhu (31)	2023	China	PCOS	15	NR	1,205	Acupuncture	Medications	NR		3.6	LH, testosterone, FSH, BMI	Low
Ruigen (60)	2016	China	PCOS	14	NR	1,617	Acupuncture	Medications	Cochrane tool, NR	NR	3.78	Pregnancy rate	Low
Ping	2020	China	PCOS	7	NR	514	Acupuncture	Medications	NR	NR	NR	Pregnancy rate	Low
Lina (52)	2018	China	PCOS	9	NR	769	Acupuncture plus TCM Acupuncture	Medications	NR	NR	NR	Pregnancy rate, LH, FSH	Moderate
Yang (30)	2023	China	PCOS	6	28.93	1,410	Acupuncture plus Moxibustion	Medications	Cochrane tool, 3/6	50	3.16	Endometrial thickness	Moderate
Li (34)	2023	China	POI	10	33.35	594	Electroacupuncture plus TCM Needle acupuncture plus TCM	HRT	Cochrane tool, 0/10	0	3.6	LH, FSH, estradiol	High
Jo (63)	2015	Republic of Korea	POI	8	35.46	620	Acupuncture	Shame acupuncture or HRT	Cochrane tool, 0/8	0	4.8	LH, FSH, estradiol	High
Wang (23)	2017	China	POI	8	Under 40	368	Acupuncture	HRT	Cochrane tool, 0/8	0	NR	LH, FSH, estradiol	Moderate
Ya-Jian	2020	China	POI	8	NR	496	Acupuncture Acupuncture plus HRT	HRT	Cochrane tool, 0/8	0	5.4	LH, FSH, estradiol	Moderate
Li	2020	China	POI	14	31.21	1,030	Acupuncture	HRT	Cochrane tool, 0/14	0	5.7	LH, FSH, estradiol	High
Yuanbo	2019	China	POI	14	NR	879	Acupuncture plus TCM	HRT	Cochrane tool, 7/14	50	3.42	LH, FSH, estradiol	Low
Runzi (54)	2016	China	POI	7	Under 40	538	Acupuncture	HRT	NR	NR	NR	LH, FSH, estradiol	Low
Tingting (45)	2018	China	POI	16	NR	1,357	Acupuncture	HRT	Cochrane tool, 0/16	0	5.43	LH, FSH, estradiol	Moderate
Xiaoyang (39)	2019	China	POI	14	NR	962	Acupuncture plus TCM Electroacupuncture plus TCM Auricular points plus TCM Acupoint catgut embedding plus TCM Moxibustion plus TCM	HRT	Cochrane tool, 0/14	0	3.85	LH, FSH, estradiol	Moderate
Meiling (51)	2018	China	POI	10	32.25	690	Needle acupuncture	HRT	Jadad score, 3/12	25	5.16	LH, FSH, estradiol	Low
Xiaojuan (35)	2022	China	POI	11	Under 40	775	Acupuncture	Shame acupuncture or HRT	Cochrane tool, 0/11	0	4.57	FSH, estradiol	Moderate
Jin-Huan (47)	2020	China	POI	16	32	1,102	Acupuncture and Moxibustion	HRT	Cochrane tool, 5/16	31	NR	LH, FSH, estradiol	Low
Xi (59)	2016	China	POI	12	34.2	863	Acupuncture Acupuncture plus HRT	HRT	NR	NR	NR	LH, FSH	Moderate
Yong (58)	2016	China	POI	9	NR	719	Acupuncture	HRT	NR	NR	NR	LH, FSH, estradiol	Low
Zhang (41)	2020	China	POI	16	32.93	1,307	Acupuncture Warm acupuncture Acupoint catgut embedding Acupuncture and Moxibustion	HRT	Cochrane tool, NR	NR	3.75	LH, FSH, estradiol	Moderate
Xiao	2017	China	POI	7	NR	512	Acupuncture plus HRT	HRT	NR	NR	5	LH, FSH, estradiol	Moderate
Long (36)	2022	China	POI	32	NR	3,231	Acupuncture and Moxibustion Acupoint catgut embedding Acupuncture plus HRT Acupoint catgut embedding plus HRT Moxibustion Acupuncture Acupuncture plus TCM	HRT	Cochrane tool, 6/32	18	4.06	FSH	Moderate

TCM, traditional Chinese medicine; NR, not reported; LH, luteinizing hormone; FSH, follicle stimulating hormone; OGTT, oral glucose tolerance test; HOMA-IR, homeostatic model assessment for insulin resistance; BMI, body mass index; HRT, hormone replacement therapy; PCOS, polycystic ovary syndrome; POI, primary ovarian insufficiency.

### Quality of the studies

Among the 20 meta-analyses on PCOS, the quality of studies was high in 4 studies, moderate in 12 studies, and low in 4 studies. Of 18 meta-analyses on POI, the study quality was rated as high in 3 studies, moderate in 10 studies, and low in 5 studies (Supplementary Table S1).

### Umbrella meta-analysis for PCOS

**Figure 2:**
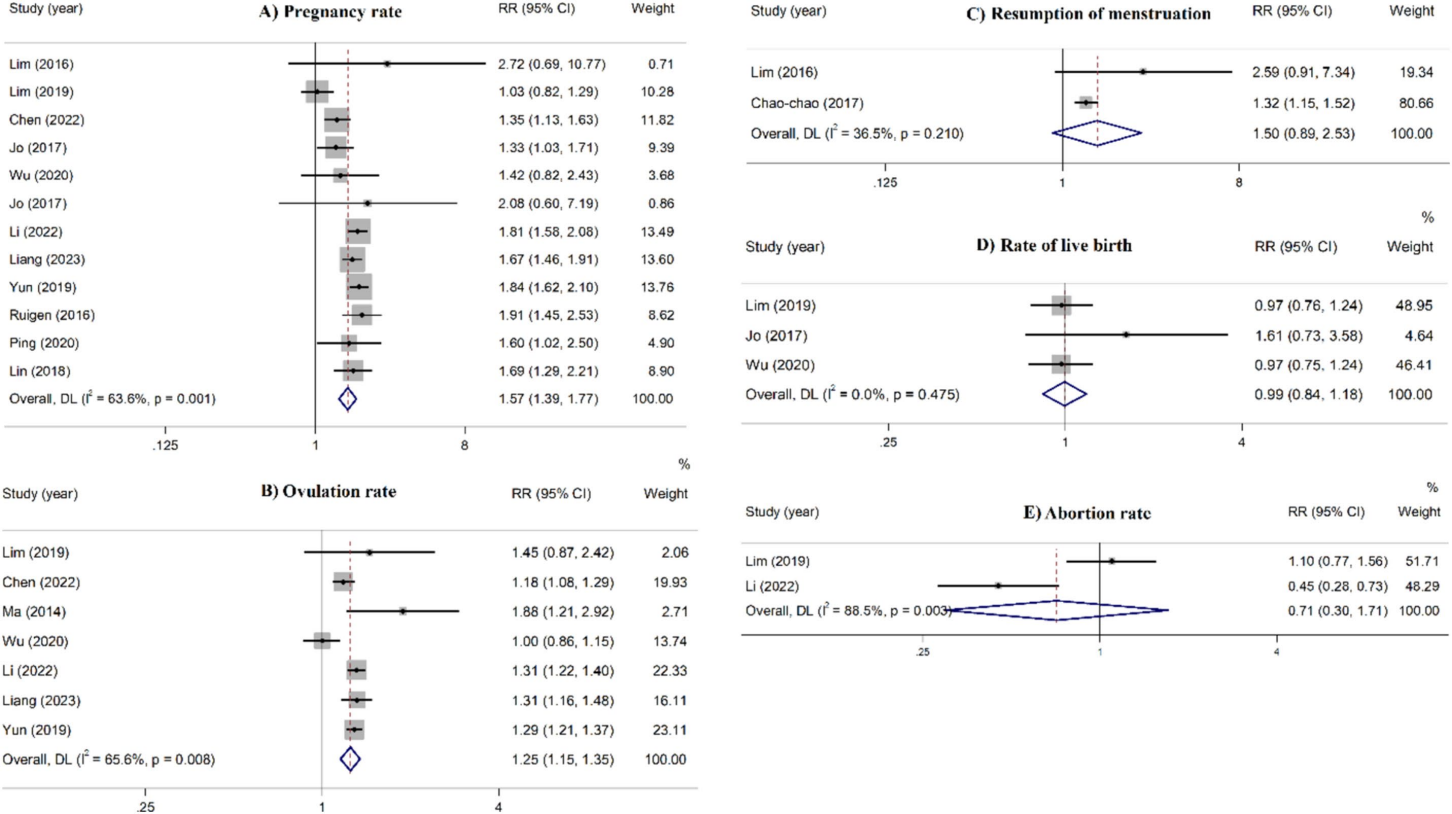
Umbrella meta-analysis for the effect of acupuncture on pregnancy-related dichotomous outcomes in patients with PCOS.

**Figure 3:**
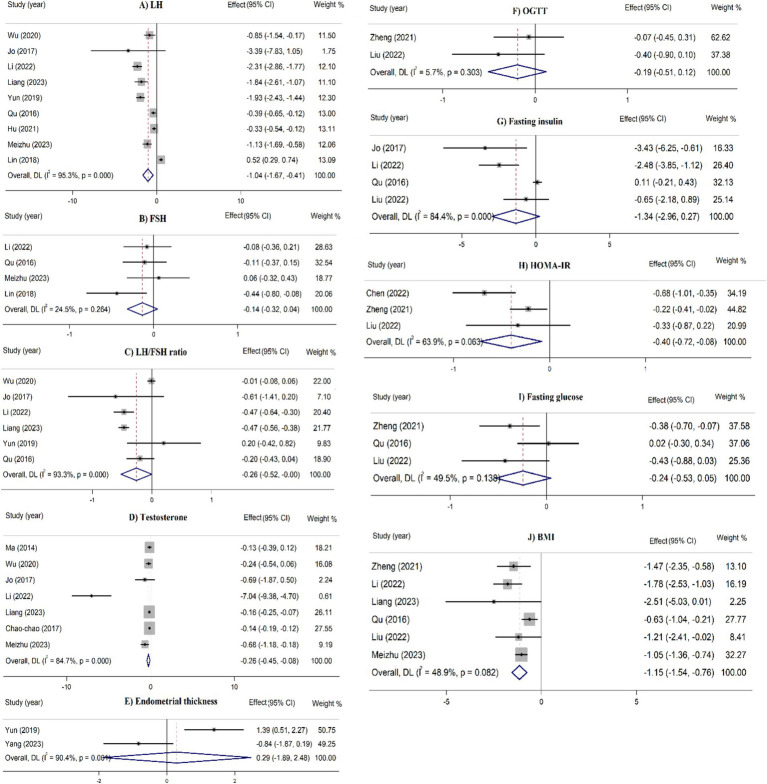
Umbrella meta-analysis for the effect of acupuncture on continuous outcomes (metabolic factors and serum hormones) in patients with PCOS FSH: follicle stimulating hormone, LH: luteinizing hormone, OGGT: oral glucose tolerance test, HOMA-IR: homeostatic model assessment for insulin resistance, BMI: body mass index.

In the overall analyses, acupuncture therapies were significantly 439 associated with a higher pregnancy rate (RR = 1.57, 95% CI: 1.39– 440 1.77), and ovulation rate (RR = 1.25, 95% CI: 1.15–1.35) (Figure 2). Moreover, acupuncture therapies significantly reduced serum levels of LH (SMD = −1.04, 95% CI: −1.67 to −0.41), LH/FSH ratio (SMD = −0.26, 95% CI: −0.52 to −0.003), testosterone levels (SMD = −0.26, 95% CI: −0.45 to −0.08), HOMA-IR (SMD = −0.40, 445, 95% CI: −0.72 to −0.08), and BMI (SMD = −1.15, 95% CI: −1.54 to 446 −0.76) (Figure 3). These effects were supported by the majority of subgroups. However, for ovulation rate and LH/FSH ratio, the treatment was effective when administered for a longer duration (≥4 months) (Table 2).

**Table 2:** Subgroup analyses for the effect of acupuncture on PCOS.

Dichotomous outcome				Test of effect	Test of heterogeneity	
		Subgroups	Studies	RR (95%CI)	*I*^2^ (%)	*P*
Pregnancy rate		Overall	12	1.57 (1.39–1.77)	63.6	0.001
	Type of intervention	Acupuncture	7	1.70 (1.42–2.02)	20.0	0.27
		Acupuncture plus medications	4	1.52 (1.23–1.88)	60.6	0.05
		Acupuncture plus moxibustion	1	1.81 (1.58–2.08)	–	–
		Acupuncture plus TCM	2	1.79 (1.51–2.11)	45.5	0.17
		Acupuncture plus medications and TCM	1	1.52 (1.25–1.85)	–	–
	Quality of studies	Low	2	1.82 (1.44–2.30)	0.0	0.51
		Moderate	6	1.58 (1.40–1.78)	51.6	0.06
		High	4	1.53 (0.96–2.43)	83.5	0.001
	Sample size	≥1,000 participants	8	1.58 (1.36–1.83)	74.2	0.001
		<1,000 participants	4	1.52 (1.28–1.80)	0.0	0.49
	Duration of treatment	NR	5	1.55 (1.27–1.89)	79.3	0.001
		≥4 months	1	1.81 (1.58–2.08)	–	–
		<4 months	5	1.56 (1.28–1.91)	22.8	0.26
Ovulation rate		Overall	7	1.25 (1.15–1.35)	65.6	0.008
	Type of intervention	Acupuncture	3	1.33 (0.84–2.09)	77.9	0.01
		Acupuncture plus medications	2	1.18 (1.11–1.27)	0.0	0.90
		Acupuncture plus moxibustion	–	1.31 (1.22–1.40)	–	–
		Acupuncture plus TCM	–	1.31 (1.16–1.48)	–	–
	Quality of studies	Low	1	1.88 (1.21–2.92)	–	–
		Moderate	4	1.20 (1.09–1.33)	74.8	0.008
		High	2	1.31 (1.23–1.40)	0.0	0.70
	Sample size	≥1,000 participants	6	1.23 (1.15–1.33)	64.9	0.01
		<1,000 participants	1	1.88 (1.21–2.92)	–	–
	Duration of treatment	NR	3	1.30 (1.23–1.37)	0.0	0.88
		≥4 months	1	1.31 (1.22–1.40)	–	–
		<4 months	3	1.19 (0.96–1.46)	76.9	0.01
Resumption of menstruation		Overall	2	1.50 (0.89–2.53)	36.5	0.21
Rate of live birth		Overall	3	0.99 (0.84–1.18)	0.0	0.47
	Type of intervention	Acupuncture	3	0.99 (0.84–1.18)	0.0	0.47
	Quality of studies	Moderate	2	1.08 (0.72–1.62)	29.5	0.23
		High	1	0.97 (0.76–1.24)	–	–
	Sample size	≥1,000 participants	2	0.97 (0.83–1.16)	0.0	0.99
		<1,000 participants	1	1.61 (0.73–3.57)	–	–
Abortion rate		Overall	2	0.71 (0.30–1.71)	88.5	0.003

Continuous outcomes		Subgroups	Studies	SMD (95%CI)	*I*^2^ (%)	*P*
LH		Overall	9	−1.04 (−1.67 to −0.41)	95.5	0.001
	Type of intervention	Acupuncture	3	−1.05 (−1.50 to −0.61)	0.0	0.49
		Acupuncture plus medications	3	−0.79 (−1.52 to −0.06)	62.8	0.06
		Acupuncture plus moxibustion	1	−2.31 (−2.86 to −1.76)	–	–
		Acupuncture plus TCM	1	−1.84 (−2.61 to −1.07)	–	–
		Acupuncture plus medications and TCM	1	−0.39 (−0.65 to −0.39)	–	–
	Quality of studies	Low	1	−1.13 (−1.69 to −0.57)	–	–
		Moderate	6	−0.75 (−1.39 to −0.11)	95.5	0.001
		High	2	−2.33 (−2.87 to −1.79)	0.0	0.63
	Sample size	≥1,000 participants	7	−1.44 (−2.20 to −0.67)	92.3	0.001
		<1,000 participants	2	0.07 (−0.82 to 0.96)	96.2	0.001
	Duration of treatment	NR	5	−0.74 (−1.44 to −0.03)	96.3	0.001
		≥4 months	1	−2.33 (−2.86 to −1.76)	-	-
		<4 months	3	−1.07 (−1.47 to −0.61)	0.0	0.47
FSH		Overall	4	−0.14 (−0.32 to 0.04)	24.5	0.26
	Type of intervention	Acupuncture	2	−0.09 (−0.46 to −0.18)	41.8	0.29
		Acupuncture plus moxibustion	1	−0.08 (−0.37 to 0.21)	1	1
		Acupuncture plus medications and TCM	1	−0.11 (−0.37 to −0.15)	1	1
	Quality of studies	Low	1	0.06 (−0.31 to 0.43)	–	–
		Moderate	2	−0.25 (−0.57 to 0.07)	52.9	0.14
		High	1	−0.08 (−0.37 to 0.21)	–	–
	Sample size	≥1,000 participants	2	−0.03 (−0.26 to 0.20)	0.0	0.56
		<1,000 participants	2	−0.25 (−0.57 to 0.07)	52.9	0.14
	Duration of treatment	NR	2	−0.25 (−0.57 to 0.07)	52.9	0.14
		≥4 months	1	−0.08 (−0.37 to 0.21)	–	–
		<4 months	1	0.06 (−0.31 to 0.43)	–	–
LH/FSH ratio		Overall	6	−0.26 (−0.52 to −0.003)	93.3	0.001
	Type of intervention	Acupuncture	2	−0.17 (−0.69 to 0.35)	52.5	0.14
		Acupuncture plus medications	1	−0.58 (−0.81 to −0.35)	–	–
		Acupuncture plus moxibustion	1	−0.47 (−0.64 to −0.30)	–	–
		Acupuncture plus TCM	1	−0.47 (−0.56 to −0.38)	–	–
		Acupuncture plus medications and TCM	1	−0.20 (−0.43 to 0.03)	–	–
	Quality of studies	Moderate	4	−0.17 (−0.48 to 0.15)	95.3	0.001
		High	2	−0.48 (−0.64 to −0.31)	0.0	0.73
	Sample size	≥1,000 participants	5	−0.28 (−0.58 to 0.03)	94.7	0.001
		<1,000 participants	1	−0.20 (−0.43 to 0.03)	–	–
	Duration of treatment	NR	3	−0.27 (−0.56 to 0.03)	76.1	0.02
		≥4 months	1	−0.47 (−0.64 to −0.30)	–	–
		<4 months	2	−0.17 (−0.69 to 0.35)	52.8	0.14
Testosterone		Overall	7	−0.26 (−0.45 to −0.08)	84.7	0.001
	Type of intervention	Acupuncture	4	−0.27 (−0.51 to −0.04)	30.5	0.23
		Acupuncture plus medications	2	−0.50 (−1.16 to 0.15)	88.9	0.003
		Acupuncture plus moxibustion	1	−7.04 (−9.38 to −4.70)	–	–
		Acupuncture plus TCM	1	−0.16 (−0.25 to −0.07)	–	–
		Acupoint catgut embedding	1	−0.14 (−0.17 to −0.11)	–	–
	Quality of studies	Low	2	−0.36 (−0.89 to 0.17)	72.9	0.05
		Moderate	3	−0.14 (−0.18 to −0.11)	0.0	0.75
		High	2	−3.78 (−0.10 to 2.44)	95.6	0.001
	Sample size	≥1,000 participants	6	−0.30 (−0.52 to −0.09)	87.2	0.001
		<1,000 participants	1	−0.13 (−0.38 to 0.12)	–	–
	Duration of treatment	NR	2	−0.14 (−0.18 to −0.11)	0.0	0.68
		≥4 months	1	−7.04 (−9.38 to −4.70)	–	–
		<4 months	4	−0.29 (−0.52 to −0.05)	0.29	0.23
Endometrial thickness		Overall	2	0.29 (−1.89 to 2.48)	90.4	0.001
Fasting plasma glucose		Overall	3	−0.24 (−0.53 to 0.05)	49.5	0.13
		Acupuncture	2	−0.24 (−0.44 to −0.05)	11.5	0.28
		Acupuncture plus medications	1	−0.40 (−1.03 to 0.23)	–	–
		Electroacupuncture	1	−0.79 (−2.18 to 0.60)	–	–
		Acupuncture plus medications and TCM	1	0.02 (−0.30 to 0.34)	–	–
OGTT		Overall	2	−0.19 (−0.51 to 0.12)	5.7	0.30
Fasting insulin		Overall	4	−1.34 (−2.96 to 0.27)	84.4	0.001
	Type of intervention	Acupuncture	2	−1.09 (−5.50 to 3.33)	83.5	0.01
		Acupuncture plus medications	2	−2.50 (−2.76 to −2.24)	0.0	0.64
		Acupuncture plus moxibustion	1	−2.48 (−3.85 to −1.11)	–	–
		Electroacupuncture	1	−1.36 (−5.47 to 2.75)		
		Acupuncture plus medications and TCM	1	0.11 (−0.21 to 0.43)	–	–
	Quality of studies	Moderate	2	0.08 (−0.23 to 0.39)	0.0	0.34
		High	2	−2.66 (−3.89 to −1.43)	0.0	0.55
	Sample size	≥1,000 participants	2	−2.66 (−3.89 to −1.43)	0.0	0.55
		<1,000 participants	2	0.08 (−0.23 to 0.39)	0.0	0.34
	Duration of treatment	NR	1	0.11 (−0.21 to 0.43)	–	–
		≥4 months	2	−3.43 (−6.25 to −0.61)	67.2	0.08
		<4 months	1	−1.60 (−3.39 to 0.19)	–	–
HOMA-IR		Overall	3	−0.40 (−0.72 to −0.08)	63.9	0.06
	Type of intervention	Acupuncture	2	−0.20 (−0.39 to −0.01)	0.0	0.50
		Acupuncture plus medications	2	−0.68 (−1.01 to −0.35)	0.0	0.64
		Electroacupuncture	1	−0.64 (−2.20 to 0.92)	–	–
	Quality of studies	Moderate	3	−0.40 (−0.72 to −0.08)	63.9	0.06
	Sample size	≥1,000 participants	1	−0.68 (−1.01 to −0.35)	–	–
		<1,000 participants	2	−0.23 (−0.42 to −0.05)	0.0	0.71
	Duration of treatment	≥4 months	2	−0.23 (−0.42 to −0.05)	0.0	0.71
		<4 months	1	−0.68 (−1.01 to −0.35)	–	–
BMI		Overall	6	−1.15 (−1.54 to −0.76)	48.9	0.08
	Type of intervention	Acupuncture	3	−1.09 (−1.38 to −0.80)	0.0	0.64
		Acupuncture plus medications	1	−1.36 (−2.06 to −0.66)	1	1
		Acupuncture plus moxibustion	1	−1.78 (−2.53 to −1.03)	–	–
		Electroacupuncture	1	−1.78 (−4.03 to 0.47)	–	–
		Acupuncture plus TCM	1	−2.51 (−5.03 to 0.01)	–	–
		Acupuncture plus medications and TCM	1	−0.63 (−1.04 to −0.22)	–	–
	Quality of studies	Low	1	−1.05 (−1.36 to −0.74)	–	–
		Moderate	4	−1.06 (−1.67 to −0.46)	39.8	0.17
		High	1	−1.78 (−2.53 to −1.03)	–	–
	Sample size	≥1,000 participants	3	−1.40 (−2.07 to −0.74)	52.4	0.12
		<1,000 participants	3	−0.96 (−1.52 to −0.39)	39.1	0.19
	Duration of treatment	NR	2	−1.14 (−2.78 to 0.50)	52.0	0.14
		≥4 months	3	−1.57 (−2.08 to −1.05)	0.0	0.70
		<4 months	1	−1.05 (−1.36 to −0.74)	–	–

RR, relative risk; SMD, standardized mean difference; TCM, traditional Chinese medicine; NR, not reported; LH, luteinizing hormone; FSH, follicle stimulating hormone; OGTT, oral glucose tolerance test; HOMA-IR, homeostatic model assessment for insulin resistance; BMI, body mass index.

No significant effect was identified on the resumption of 451 menstruation, rate of live birth, abortion rate, FSH levels, endometrial 452 thickness, FPG, OGTT, and fasting insulin in the overall analyses 453 (Figures 2, 3). However, in the stratified analyses, patients who received acupuncture only or acupuncture plus other therapies had a lower FSH levels. Treatment with acupuncture was also associated with a lower FPG levels. Furthermore, acupuncture plus medications as well as acupuncture plus moxibustion were linked to a lower fasting insulin in high quality studies and studies with ≥1,000 participants.

### Umbrella meta-analysis for POI

**Figure 4:**
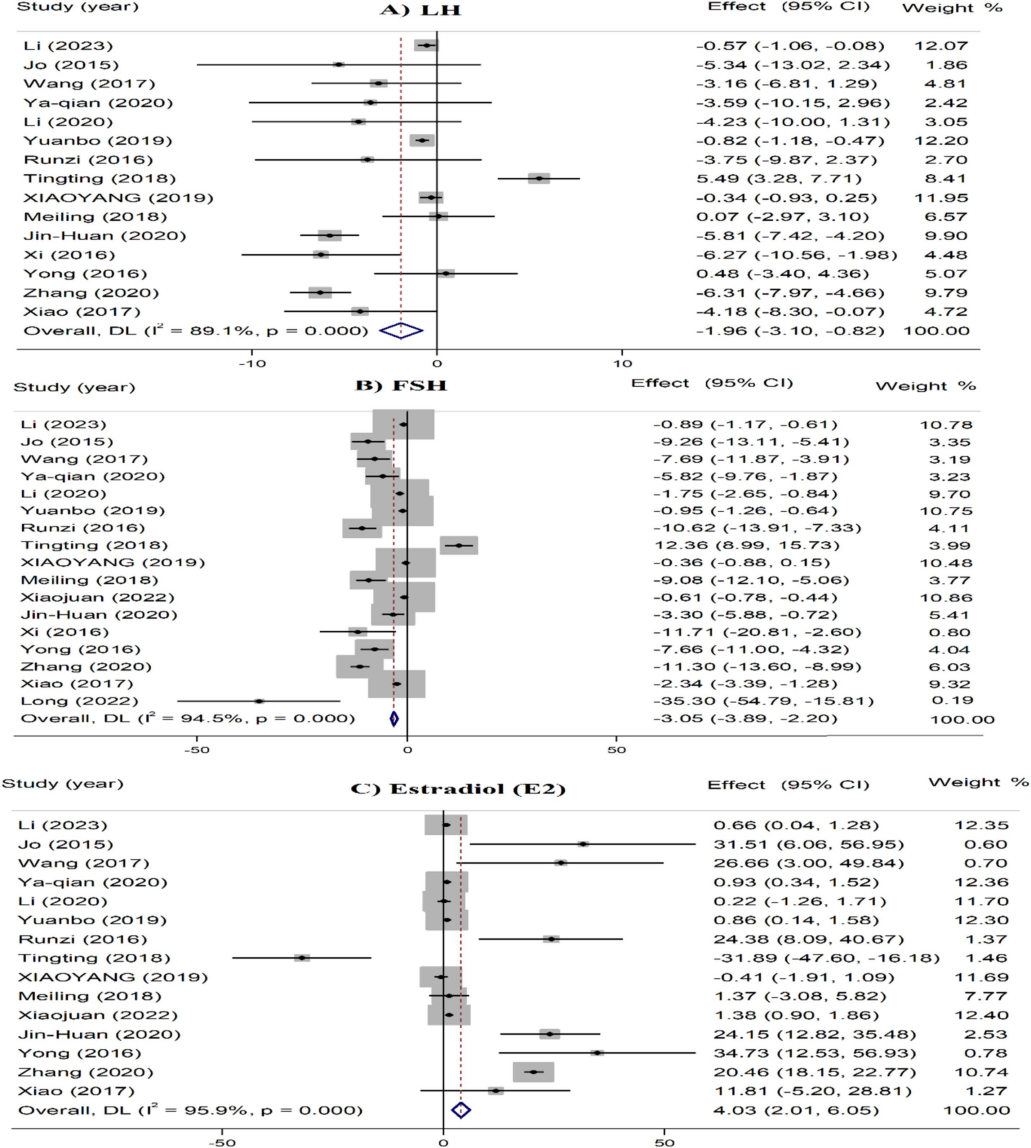
Umbrella meta-analysis for the effect of acupuncture on various outcomes in patients with POF. FSH, follicle stimulating hormone; LH, luteinizing hormone.

In the overall analyses, acupuncture interventions were significantly linked to the reduced serum levels of LH (SMD = −1.96, 95% CI: −3.10 to −0.82) and FSH (SMD = −3.05, 95% CI: −3.89 to −2.20), and increased levels of estradiol (SMD = 4.03, 95% CI: 2.01 to 6.05) (Figure 4).

In the subgroup analysis, acupuncture plus TCM, electroacupuncture plus TCM, and acupuncture plus moxibustion remarkably reduced serum LH levels. For FSH, acupuncture, acupuncture plus TCM, acupuncture plus HRT, needle acupuncture, needle acupuncture plus TCM, electroacupuncture plus TCM, and auricular points plus TCM were linked to reduced serum FSH. Regarding estradiol, acupuncture only, needle acupuncture plus TCM, auricular points plus TCM, acupoint catgut embedding plus TCM, and moxibustion plus TCM were linked to increased serum estradiol. For all outcomes, the significant effects were observed in studies with smaller sample sizes (<1,000 participants) and shorter treatment durations (<5 months) (Table 3).

**Table 3:** Subgroup analyses for the effect of acupuncture on premature ovary inefficiency.

Continuous outcomes		Subgroups	Studies	SMD (95%CI)	*I*^2^ (%)	*P*
LH		Overall	15	−1.96 (−3.10 to −0.82)	89.1	0.001
	Type of intervention	Acupuncture	8	−2.71 (−6.86 to 1.44)	90.9	0.001
		Acupuncture plus TCM	2	−0.64 (−1.10 to −0.18)	46.4	0.17
		Acupuncture plus HRT	2	−6.80 (−13.84 to 0.23)	54.2	0.14
		Needle acupuncture	1	0.07 (−2.97 to 3.11)	–	–
		Needle acupuncture plus TCM	1	−0.22 (−0.48 to 0.04)	–	–
		Electroacupuncture plus TCM	2	−1.29 (−1.60 to −0.98)	0.0	0.99
		Auricular points plus TCM	1	−1.46 (−3.26 to 0.34)	–	–
		Acupoint catgut embedding plus TCM	1	−0.40 (−2.84 to 2.06)	–	–
		Warm acupuncture	1	0.32 (−5.64 to 6.28)	–	–
		Acupuncture and moxibustion	1	−5.81 (−7.42 to −4.20)	–	–
		Moxibustion plus TCM	1	0.84 (−0.47 to 1.23)	–	–
	Quality of studies	Low	5	−1.97 (−4.77 to 0.83)	89.2	0.001
		Moderate	7	−2.41 (−5.65 to 0.84)	93.1	0.001
		High	3	−1.78 (−4.60 to 1.01)	34.6	0.21
	Sample size	≥1,000 participants	4	−2.68 (−8.38 to 3.03)	96.4	0.001
		<1,000 participants	11	−0.80 (−1.31 to −0.28)	39.3	0.08
	Duration of treatment	NR	5	−3.91 (−6.46 to −1.36)	59.3	0.04
		≥5 months	5	−0.88 (−5.32 to 3.55)	85.1	0.001
		<5 months	5	−1.68 (−2.81 to −0.55)	91.5	0.001
FSH		Overall	17	−3.05 (−3.89 to −2.20)	94.5	0.001
	Type of intervention	Acupuncture	11	−4.84 (−7.60 to −2.08)	96.2	0.001
		Acupuncture plus TCM	3	−0.70 (−1.31 to −0.09)	61.1	0.07
		Acupuncture plus HRT	3	−5.79 (−11.19 to −0.40)	67.1	0.05
		Needle acupuncture	1	−9.08 (−12.60 to −5.56)	–	–
		Needle acupuncture plus TCM	1	−0.62 (−1.02 to −0.22)	–	–
		Electroacupuncture plus TCM	2	−1.17 (−1.38 to −0.97)	0.0	0.88
		Auricular points plus TCM	1	−2.19 (−2.97 to −1.41)	–	–
		Acupoint catgut embedding	2	−0.77 (−37.14 to 35.60)	95.5	0.001
		Acupoint catgut embedding plus TCM	1	−0.72 (−2.13 to 0.69)	–	–
		Acupoint catgut embedding plus HRT	1	−10.05 (−22.09 to 1.97)	–	–
		Warm acupuncture	1	2.50 (−5.67 to 10.67)	–	–
		Acupuncture and moxibustion	3	3.15 (−4.52 to 10.83)	87.0	0.002
		Moxibustion	1	2.32 (−5.84 to 10.48)	–	–
		Moxibustion plus TCM	1	−0.02 (−0.38 to 0.34)	–	–
	Quality of studies	Low	5	−6.15 (−10.39 to −1.90)	94.3	0.001
		Moderate	9	−2.75 (−4.61 to −0.90)	95.5	0.001
		High	3	−2.55 (−4.44 to −0.65)	90.4	0.001
	Sample size	≥1,000 participants	5	−3.87 (−10.72 to 2.98)	97.2	0.001
		<1,000 participants	12	−2.50 (−3.23 to −1.77)	91.6	0.001
	Duration of treatment	NR	5	−7.59 (−10.74 to −4.44)	71.0	0.008
		≥5 months	5	−1.30 (−5.05 to 2.44)	95.5	0.001
		<5 months	7	−1.96 (−2.79 to −1.14)	94.9	0.001
Estradiol (E2)		Overall	15	4.03 (2.01 to 6.05)	95.5	0.001
	Type of intervention	Acupuncture	9	6.59 (2.38 to 10.80)	97.4	0.001
		Acupuncture plus TCM	2	0.40 (−0.79 to 1.60)	55.3	0.13
		Acupuncture plus HRT	2	3.00 (−4.29 to 10.28)	30.1	0.23
		Needle acupuncture	1	1.37 (−3.08 to 5.82)	–	–
		Needle acupuncture plus TCM	1	0.60 (0.14 to 1.06)	–	–
		Electroacupuncture plus TCM	2	0.63 (−0.40 to 1.66)	0.0	0.87
		Auricular points plus TCM	1	1.63 (1.21 to 2.05)	–	–
		Acupoint catgut embedding	1	8.06 (−10.88 to −5.24)	–	–
		Acupoint catgut embedding plus TCM	1	1.32 (0.69 to 1.95)	–	–
		Warm acupuncture	1	4.60 (−24.55 to 15.35)	–	–
		Acupuncture and moxibustion	2	13.35 (−7.32 to 34.02)	87.4	0.05
		Moxibustion plus TCM	1	0.64 (0.27 to 1.01)	–	–
	Quality of studies	Low	5	11.99 (3.67 to 20.32)	87.9	0.001
		Moderate	7	4.37 (0.51 to 8.31)	97.9	0.001
		High	3	0.62 (−1.15 to 2.39)	66.4	0.05
	Sample size	≥1,000 participants	4	4.64 (−10.55 to 19.84)	98.8	0.001
		<1,000 participants	11	0.98 (0.17 to 1.80)	71.9	0.0.001
	Duration of treatment	NR	4	25.90 (17.84 to 33.95)	0.0	0.86
		≥5 months	5	0.14 (−2.50 to 2.78)	79.0	0.001
		<5 months	6	4.65 (1.68 to 7.62)	98.2	0.001

SMD, standardized mean difference; TCM, traditional Chinese medicine; NR, not reported; HRT, hormone replacement therapy; LH, luteinizing hormone; FSH, follicle stimulating hormone.

### Heterogeneity, meta-regression, sensitivity analysis, and publication bias

**Figure 5:**
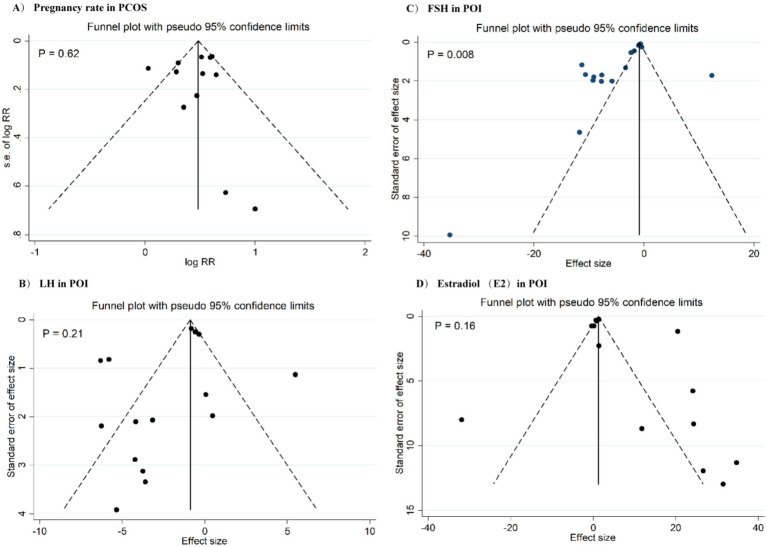
Funnel plots for publication bias for pregnancy rate in PCOS **(A)**, LH in POF **(B)**, FSH in POF **(C)**, and estradiol in POF **(D)**.

There was a significant heterogeneity across the studies for most outcomes (Tables 2, 3). Meta-regression analysis indicated that the results were not significantly affected by the proportion of RCTs with low ROB in each meta-analysis, publication year, and the number of effect sizes for each outcomes. In the sensitivity analyses, the pooled effect sizes for HOMA-IR, LH, fasting insulin, and FPG were significantly affected by the individual studies. A significant publication bias was detected for FSH levels in POI patients (Figure 5).

## Discussion

This umbrella meta-analysis aimed to explore the impact of acupuncture on the clinical outcomes of patients with PCOS and POI. The analysis revealed that acupuncture significantly improved pregnancy rate and ovulation rate, and favorably affected serum levels of LH, testosterone, LH/FSH ratio, HOMA-IR, and BMI in PCOS patients. Moreover, there was weak evidence for the positive effects of acupuncture on other hormonal and metabolic parameters including, FSH, FPG, and fasting insulin. Additionally, we found that acupuncture, when compared to standard care (HRT) resulted in a reduction in LH and FSH levels, alongside an increase in serum estradiol levels.

Acupoint stimulation and TCM are frequently used in several Asian countries as complementary and alternative therapies for POI and PCOS. Although acupuncture has shown potential in treating both POI and PCOS, the lack of high-quality evidence-based studies hinders drawing any firm conclusion on whether acupuncture affects the clinical outcomes of women with POI and PCOS. Recent meta-analyses have yielded contradictory results. While Chen et al. (24) and Liang et al. (33) showed an improvement in the pregnancy rate, other meta-analyses found no significant change (44, 50). The results for hormonal and metabolic changes are also inconsistent (23, 37). The heterogeneity in the results of the previous studies could be due to the differences in the treatment protocol, sample size, duration of the intervention, and disease phenotype. Our analysis revealed that acupuncture increases pregnancy rate and improves metabolic and hormonal imbalances in these patients. Currently, there is no sufficient data to determine the optimal acupuncture treatment in patients with POI and PCOS due to the limited number of available studies. In addition, several contributing factors, including acupoint specificity and selection, stimulation methods, the number of sessions, and the practitioner’s expertise could affect the results (55). Additional studies are recommended to investigate the optimal acupuncture protocols for POI/PCOS.

The present study found that the duration of acupuncture treatment significantly impacted outcome. For some PCOS outcomes and for all outcomes of POI, acupuncture improved the clinical outcomes when administered for a longer duration, but not when used for a shorter period. The explanation for this finding is that the longer duration of acupuncture treatment allows for a more sustained and profound impact on the neuro-endocrine system and the ovarian environment, especially in cases of POI where the ovarian function is compromised. In contrast, shorter durations of acupuncture treatment may not be sufficient to achieve the same level of improvement in ovarian function. This could be due to the limited time for acupuncture to stimulate the neuro-endocrine system and promote follicular development.

The clinical utility of our findings is that acupuncture can be a valuable adjunctive therapy for managing POI and PCOS, particularly in cases where conventional treatments are ineffective or have significant side effects. Furthermore, the improvement in hormonal and metabolic imbalances, such as insulin resistance, obesity, and hyperandrogenism, indicates that acupuncture can have a positive impact on the underlying pathophysiology of PCOS (67). This can lead to better management of symptoms, enhanced life quality, and a decrease in the odds of long-term complications, such as cardiovascular disease and diabetes (68).

Acupuncture could improve PCOS through several key mechanisms, including neuroendocrine regulation, hormone regulation, stem cell regulation, follicular development and maturation, gene expression, and improving the hemodynamics of ovary and uterus (69, 70). Acupuncture regulates the neuroendocrine system, which plays an essential role in the progression of PCOS. This includes the hypothalamus-pituitary-ovary axis, affecting the secretion of ovarian-related hormones, such as estradiol, FSH, and LH, which are essential for normal ovarian function and menstruation cycles (71). Studies have shown that acupuncture decreases cortisol levels and triggers the production of pituitary beta-endorphins, which exert a tonic inhibitory influence on the gonadotropin-releasing hormone pulse generator and pituitary LH secretion, which is a potential for acupuncture to alleviate ovulatory dysfunction in PCOS (44, 46). Acupuncture may also regulate stem cell populations in the ovaries, which are important for follicular development and maturation (72). Furthermore, animal studies have indicated a significant increase in ovarian tissue and endometrium thickness, an increase in normal glands and blood vessels, a rise in the number of antral follicles, and a decrease in interstitial cells after treatment with acupuncture (46).

Acupuncture has been shown to affect specific genes that play an important role in treating PCOS. Acupuncture improves follicular development and maturation by inhibiting granulosa cell apoptosis and promoting the proliferation of granulosa cells. This is achieved through the downregulation of the MEG3 gene in ovarian granulosa cells, which leads to decreased expression of miR-21-3p. This pathway contributes to the development of PCOS and is related to ovulatory dysfunction and abnormal follicular development (72). Acupuncture also promotes Bcl-2 gene expression and inhibits Bax gene expression (71), which are involved in the pathogenesis of PCOS. Acupuncture also upregulates PLA2G4A and downregulates miR-32-3p, potentially enhancing ovulation and improving endocrine function, especially in PCOS patients with diabetes (73). A study found that electroacupuncture altered the expression of metabolic-related genes, particularly FOSB and LOC100128899. It also enhanced the expression of LXR/RX while reversing PPARγ and ADIPOR2 expressions (74). Acupuncture has been found to improve insulin sensitivity by increasing the expression of insulin receptor substrate 1 (IRS1) and IRS2 in the endometrial tissue of PCOS cases. This may contribute to better insulin sensitivity in the endometrium (75). Gene expression analysis revealed that electroacupuncture upregulated the IRS-1/PI3K/GLUT4 signaling pathway in PCOS patients, which may enhance oocyte quality and embryonic development potential (76). Overall, these findings suggest that acupuncture alters gene expression in ways that may alleviate PCOS symptoms, indicating its potential as an effective therapeutic option.

In the present study, acupuncture decreased FSH and LH levels but increased estradiol levels in POI patients. Acupuncture may improve hormone imbalance in patients with POI through improvement of ovarian function, modulation of the hypothalamic–pituitary-ovarian (HPO) Axis, and reduction of stress (77, 78). The treatment may enhance ovarian blood flow and function, which can lead to improved ovarian response and hormone production (79). Acupuncture can influence the HPO axis, potentially enhancing the secretion of gonadotropin-releasing hormone (GnRH), which regulates FSH and LH release (80). By alleviating stress and anxiety, acupuncture may help reduce cortisol levels, which can negatively impact reproductive hormones and overall hormonal balance (81).

This study is the first umbrella meta-analysis examining the clinical efficacy of acupuncture on patients with PCOS and POI. The strengths of the study includes a high number of analyzed studies with a large sample size, examination of various important clinical outcomes, and subgroup and meta-regression analyses to assess the origins of heterogeneity. Moreover, the efficacy of various acupuncture therapies were analyzed compared to their parallel controls to investigate their specific impacts. Some limitation of this study should be acknowledged. First, a remarkable heterogeneity was revealed in most analyses, limiting the generalizability of the results. Subgroup analyses found that differences in treatment protocol, follow-up duration, sample size, and quality of studies contributed to the observed heterogeneity across the studies. The heterogeneity was not originated from the variability in the age of participants and the proportion of RCTs with low ROB in included studies. We also applied a random effects approach for analyses to decrease the effect of the heterogeneity on the pooled effect sizes. Second, publication bias was significant for some outcomes. Despite conducting searches without language restrictions across various databases, some small studies might be ignored, resulting to the significant small-study effect. Nevertheless, the trim-and-fill analysis indicated a minimal impact of publication bias on the combined estimates, suggesting the reliability of the results. Third, among the studies, different acupuncture strategies with different combinations with other therapies were used, which can influence the findings. To evaluate the distinct effects of different acupuncture treatments on the outcomes, we conducted subgroup analyses based on the type of treatment. The heterogeneity in acupoint selection and acupuncture techniques, may limit the reliability of our conclusions. Therefore, we recommend the need for standardized protocols in future studies to improve comparability and reliability in acupuncture research for both PCOS and POI. Moreover, the presence of overlapping studies among systematic reviews is an inherent limitation of umbrella meta-analyses that can affect the reliability of our findings. This represents another limitation of our study. Lastly, the sensitivity analysis identified that the pooled estimates for HOMA-IR, LH/FSH ratio, fasting insulin, and fasting glucose in PCOS patients were influenced by individual studies, highlighting that these results should be interpreted with caution. Further studies are required to validate our findings. Furthermore, given the limited number of studies for some subgroups, the results of should be interpreted with caution as there is a likely risk of false negative results.

In conclusion, this umbrella meta-analysis suggests that acupuncture-related interventions are potential alternative therapies for patients with PCOS and POI. Acupuncture not only increased the pregnancy rate and ovulation rate in PCOS patients, but also showed favorable effects on hormonal and metabolic parameters. Acupuncture therapy also improved hormonal changes in women with POI. Given the high risk of bias in existing RCTs and a notable heterogeneity across the meta-analyses of RCTs, large-scale RCTs with robust methodological standards are required to gain a better understanding of the role of acupuncture in these patients.

## Data Availability

The original contributions presented in the study are included in the article/Supplementary material, further inquiries can be directed to the corresponding author.
