# Supplementary material for: The effects of acupuncture on patients with premature ovarian insufficiency and polycystic ovary syndrome: an umbrella review of systematic reviews and meta-analyses
Source: Front Med (Lausanne). 2024 Nov 25;11:1471243. doi: 10.3389/fmed.2024.1471243 (PMC11627218; doi:10.3389/fmed.2024.1471243)
Supplement: Supplementary file 1 [file Table_1.DOCX]

| Study | Q1 | Q2 | Q3 | Q4 | Q5 | Q6 | Q7 | Q8 | Q9 | Q10 | Q11 | Q12 | Q13 | Q14 | Q15 | Q16 | Quality |
| --- | --- | --- | --- | --- | --- | --- | --- | --- | --- | --- | --- | --- | --- | --- | --- | --- | --- |
| Lim et al., 2016 | Yes | Yes | Yes | Partial  Yes | Yes | Yes | Yes | Yes | Yes | No | Yes | Yes | Yes | Yes | Yes | No | High |
| Lim et al. 2019 | Yes | Yes | Yes | Yes | Yes | Yes | Yes | Yes | Yes | Yes | Yes | Yes | Yes | Yes | Yes | No | High |
| Chen et al., 2022 | No | Yes | Yes | Partial  Yes | Yes | Yes | No | No | Yes | Yes | Yes | No | Yes | Yes | No | Yes | Moderate |
| Li et al., 2023 | Yes | Yes | Yes | Yes | No | Yes | Partial Yes | Yes | Yes | Yes | Yes | Yes | Yes | Yes | No | Yes | High |
| Ma et al., 2014 | Yes | No | Yes | Partial Yes | No | No | No | Partial Yes | Yes | No | Yes | Yes | Yes | Yes | No | No | Low |
| Jo et al. ,2015 | Yes | No | Yes | Yes | Yes | Yes | Yes | Yes | Yes | Yes | Yes | No | Yes | Yes | No | Yes | High |
| Jo et al. ,2017 | Yes | No | Yes | Partial  Yes | Yes | Yes | No | Partial Yes | Yes | No | Yes | Yes | No | Yes | No | No | Low |
| Wang et al., 2017 | Yes | No | Yes | Partial  Yes | No | Yes | No | Yes | Yes | Yes | Yes | No | Yes | Yes | Yes | Yes | Moderate |
| Ya-qian et al., 2020 | Yes | No | Yes | Partial  Yes | Yes | Yes | No | Yes | Yes | No | Yes | No | Yes | No | Yes | No | Moderate |
| Zheng et al., 2021 | Yes | No | Yes | Partial Yes | Yes | Yes | No | Partial Yes | Yes | No | Yes | Yes | Yes | Yes | No | Yes | Moderate |
| Wu et al., 2020 | Yes | Yes | Yes | Partial  Yes | Yes | Yes | No | Partial Yes | Yes | No | Yes | Yes | Yes | Yes | No | Yes | Moderate |
| Jo et al. ,2017 | Yes | No | Yes | Yes | Yes | Yes | Yes | Yes | Yes | No | Yes | Yes | Yes | Yes | No | Yes | High |
| Li et al., 2020 | Yes | Yes | Yes | Partial  Yes | Yes | Yes | Yes | Yes | Yes | Yes | Yes | No | Yes | Yes | Yes | Yes | High |
| Li et al., 2022 | Yes | Yes | Yes | Yes | Yes | Yes | Yes | Yes | Yes | Yes | Yes | Yes | Yes | Yes | Yes | Yes | High |
| Liang et al., 2023 | Yes | No | Yes | Partial  Yes | Yes | Yes | No | Yes | Yes | Yes | Yes | No | Yes | Yes | Yes | Yes | Moderate |
| Yun et al., 2019 | Yes | No | Yes | No | No | Yes | No | Yes | Yes | Yes | Yes | No | Yes | Yes | Yes | No | Moderate |
| Qu et al., 2016 | Yes | No | Yes | Partial  Yes | Yes | Yes | No | Partial Yes | Yes | No | Yes | Yes | Yes | Yes | No | Yes | Moderate |
| Hu et al., 2021 | No | No | Yes | Partial Yes | Yes | Yes | Yes | Yes | Yes | Yes | Yes | No | Yes | Yes | Yes | Yes | Moderate |
| Liu et al., 2022 | Yes | Yes | Yes | Yes | Yes | Yes | Yes | Yes | Yes | No | Yes | No | No | No | No | No | Moderate |
| Runzi et al., 2016 | Yes | No | Yes | No | No | No | Partial Yes | Yes | NO | No | Yes | No | Yes | No | No | No | Low |
| Yuanbo et al., 2019 | Yes | No | Yes | Partial Yes | No | No | No | No | Yes | No | Yes | No | Yes | Yes | No | No | Low |
| Tingting et al., 2018 | Yes | No | Yes | Partial Yes | No | Yes | No | Yes | Yes | No | Yes | No | Yes | Yes | Yes | No | Moderate |
| XIAOYANG et al., 2019 | Yes | No | Yes | Partial Yes | Yes | Yes | No | No | Yes | No | Yes | No | Yes | Yes | Yes | No | Moderate |
| Meiling et al., 2018 | Yes | No | Yes | Partial Yes | No | Yes | No | No | Yes | No | Partial Yes | No | No | Yes | Yes | No | Low |
| Chao-chao et al., 2017 | Yes | No | Yes | Partial Yes | No | Yes | Yes | Yes | Yes | No | Yes | Yes | Yes | Yes | No | Yes | Moderate |
| Meizhu et al., 2023 | Yes | No | Yes | Partial Yes | Yes | Yes | Yes | No | NO | No | Yes | No | No | No | No | No | Low |
| Ruigen et al., 2016 | Yes | No | Yes | Partial Yes | Yes | Yes | No | No | Yes | No | No | No | No | Yes | Yes | Yes | Low |
| Xiaojuan et al., 2022 | Yes | No | Yes | Yes | Yes | Yes | Yes | Partial Yes | Yes | No | Yes | No | No | Yes | Yes | No | Moderate |
| Ping et al., 2020 | Yes | No | Yes | Partial Yes | Yes | Yes | No | No | NO | No | Yes | No | Yes | No | Yes | No | Low |
| Jin-Huan et al., 2020 | Yes | No | Yes | Partial Yes | No | Yes | No | Partial Yes | Yes | No | Yes | No | Yes | Yes | Yes | No | Low |
| Xi et al., 2016 | Yes | No | Yes | Partial Yes | Yes | Yes | No | No | Yes | No | Yes | No | Yes | Yes | Yes | No | Moderate |
| Lin et al., 2018 | Yes | No | Yes | Partial Yes | No | Yes | Yes | Yes | Yes | No | Yes | No | Yes | Yes | Yes | No | Moderate |
| Yong et al., 2016 | Yes | No | Yes | Partial Yes | No | No | No | No | Yes | No | Yes | No | Yes | No | Yes | No | Low |
| Zhang et al., 2020 | Yes | Yes | Yes | Partial Yes | No | Yes | Yes | Partial Yes | Yes | Yes | Yes | No | Yes | Yes | No | Yes | Moderate |
| Xiao et al., 2017 | Yes | No | Yes | Partial Yes | Yes | Yes | Yes | Yes | Yes | No | Yes | No | Yes | No | Yes | No | Moderate |
| Long et al., 2022 | Yes | No | Yes | Partial Yes | Yes | Yes | No | Yes | Yes | No | Yes | No | Yes | No | Yes | No | Moderate |
| Yang et al., 2023 | No | No | Yes | Partial Yes | Yes | Yes | Yes | Yes | Yes | Yes | No | No | No | Yes | Yes | Yes | Moderate |
| Yang et al., 2017 | Yes | No | Yes | Partial Yes | No | Yes | Yes | Yes | Yes | No | Yes | No | Yes | Yes | Yes | No | Moderate |

Table S1. Results of assessment of the methodological quality of meta-analysis.

1. Did the research questions and inclusion criteria for the review include the components of PICO? 2. Did the report of the review contain an explicit statement that the review methods were established prior to the conduct of the review and did the report justify any significant deviations from the protocol? 3. Did the review authors explain their selection of the study designs for inclusion in the review? 4. Did the review authors use a comprehensive literature search strategy? 5. Did the review authors perform study selection in duplicate? 6. Did the review authors perform data extraction in duplicate? 7. Did the review authors provide a list of excluded studies and justify the exclusions? 8. Did the review authors describe the included studies in adequate detail? 9. Did the review authors use a satisfactory technique for assessing the risk of bias (RoB) in individual studies that were included in the review? 10. Did the review authors report on the sources of funding for the studies included in the review? 11. If meta-analysis was performed, did the review authors use appropriate methods for statistical combination of results? 12. If meta-analysis was performed, did the review authors assess the potential impact of RoB in individual studies on the results of the meta-analysis or other evidence synthesis? 13. Did the review authors account for RoB in individual studies when interpreting/discussing the results of the review? 14. Did the review authors provide a satisfactory explanation for, and discussion of, any heterogeneity observed in the results of the review? 15. If they performed quantitative synthesis, did the review authors carry out an adequate investigation of publication bias (small study bias) and discuss its likely impact on the results of the review? 16. Did the review authors report any potential sources of conflict of interest, including any funding they received for conducting the review? Each question was answered with “Yes”, “Partial Yes” or “No”. When no meta-analysis was done, question 11, 12, and 15 were answered with “No meta-analysis conducted.”
